# The Vaccination of Indians

**Published:** 1860-08

**Authors:** 


*The Vaccination of Indians.*—We learn from the "National Intelligencer" that one of the Senate's amendments to the Indian Appropriation bill provides, to a limited extent, for the continuation of vaccination among the Indians, recently suspended in consequence of the appropriation having run out. This horrible disease has carried off thousands of the "red men of the forest." By reference to the reports of the office of Indian Affairs for the year 1837–8, we learn that the small-pox swept away whole tribes of these unfortunate people, and that of the Sioux alone 17,200 died of the disease. More recently, in the year 1853, nearly 12,000 of the confederated bands of the Sioux and Omahas, died with the same terrible malady. In 1857, four hundred of the Pawnees died from its effects.—*Boston Med. Jour.*

